# Influenzal Sinus Disease

**Published:** 1918-09

**Authors:** 


***Influenzal Sinus Disease and its Relation to Epidemic Influenza.*** By. H. E. Robertson, Major, M. R. C. *Journal A.M.A.*, May 25, 1918.

In the summary of this important article the author condenses the facts in the following paragraphs:

1. Epidemics of respiratory influenza (purulent tracheobronchitis) have been fairly severe in both the American and British Expeditionary Forces.

2. In the investigation of cases, both clinically and at *post mortem*, little attention in the past has been given the question of accompanying sinus disease.

3. Of 8 fatal cases of purulent tracheobronchitis due to the influenza bacillus, all but one showed involvement of one or more of the sinuses at the base of the skull by inflammatory processes, probably in all cases directly due to the invasion of these sinuses by the influenza bacillus.

4. In 6 patients that died from other apparently independent infection, the sinuses showed influenzal inflammation.

5. Of 2 patients dying from accidentally received injuries, both harbored in their sinuses lesions giving pure cultures of B. influenzae.

6. Appropriate treatment of the sinuses in patients suffering from influenza often served to relieve the symptoms and apparently to hasten convalescence.

7. Investigation of the sinuses during epidemics of influenza is strongly recommended and urged not only on therapeutic but also on prophylactic grounds.

